# Reconsidering Ondansetron for Smoking Cessation: A Call for Renewed Investigation

**DOI:** 10.1002/prp2.70195

**Published:** 2025-11-16

**Authors:** Amanda Tam, Zoya Munsar, Douglas J. Opler

**Affiliations:** ^1^ Department of Psychiatry Rutgers New Jersey Medical School Newark New Jersey USA

We propose that ondansetron, a 5‐HT3 receptor antagonist traditionally used for nausea, warrants renewed consideration as a potential novel treatment for smoking cessation given both inadequate prior investigation and new findings. Cigarette smoking continues to be the leading cause of preventable death worldwide, and although current first‐line treatments, including nicotine replacement therapy, bupropion, varenicline, and cognitive‐behavioral interventions, can be effective, maintaining long‐term abstinence remains a challenge [1]. Relapse rates are high, with one study reporting abstinence rates as low as 8%–25% and another reporting rates up to 40% 1 year after treatment completion despite initial success [1, 2]. Thus, there remains a need for more effective and better‐tolerated therapeutic options.

While ondansetron has previously been explored for smoking cessation, the evidence base is limited. In the 1990s, multiple animal studies supported its potential utility: Costall et al. demonstrated that intraperitoneal ondansetron (0.01 mg/kg BID) reduced behavioral suppression following nicotine withdrawal in rodents [3]. Similarly, Suzuki et al. found that ondansetron dose‐dependently blocked place aversion in nicotine‐dependent rats, suggesting that it may alleviate the negative affective symptoms of nicotine withdrawal [4].

On the other hand, prior research in humans is less convincing. Two small studies have previously evaluated ondansetron for smoking cessation in humans with negative findings. Zacny et al. conducted two placebo‐controlled experiments: the first used a crossover design (*n* = 7), testing doses up to 0.45 mg/kg, while the second used a new group of participants (*n* = 7) with even lower doses (up to 0.04 mg/kg) [5]. They reported that neither experiment showed significant effects on cigarette consumption, nicotine exposure, or mood. Additionally, West and Hajek reported that a dose of 0.25 mg BID of ondansetron did not reduce withdrawal symptoms or improve abstinence rates over 4 weeks in a sample of 111 smokers [6].

Notably, these early human studies had several methodological flaws that may have obscured potential benefits of ondansetron for smoking cessation. Both experiments by Zacny et al. were constrained by small sample sizes, short treatment duration (only 24 h), and lack of long‐term follow‐up. Participants were not motivated to quit smoking and were treated as inpatients, which is not the typical environment in which individuals would attempt to quit smoking [7]. Outcomes such as expired carbon monoxide and plasma nicotine levels were not statistically analyzed, and mood effects were noted without any supporting data or analyses [7]. In addition, because the optimal dose of ondansetron remains unknown, the doses required to obtain a therapeutic response may be substantially higher than those tested in these studies. West and Hajek employed a relatively low dose of ondansetron and, despite a stronger study design, did not demonstrate efficacy. A summary of these early studies, including their methodological limitations, is provided in Table 1.

**TABLE 1:** Summary of early studies from the 1990s evaluating ondansetron for smoking cessation, including study type, sample size, dosing and duration, key findings, and methodological flaws.

Summary of early studies evaluating ondansetron for smoking cessation	
Costall et al. [3]	Suzuki et al. [4]
Study Type: Preclinical (mice: light/dark test; rats: social interaction)	Study Type: Preclinical (rats; conditioned place preference paradigm)
*N*: Mice: *n* = 8/group; Rats: *n* = 6 pairs/group	*N*: ~8–16 per group
Dose & Duration: Ondansetron 0.01–1.0 μg/kg IP acute; 0.01 mg/kg IP b.i.d. for 7–14 days; withdrawal tested	Dose & Duration: Nicotine dependence: 9 mg/kg/day for 7 days (osmotic minipump); Mecamylamine 1 mg/kg s.c. precipitated withdrawal; Ondansetron 0.01–0.1 mg/kg s.c. 30 min before mecamylamine
Key Findings: Ondansetron reduced behavioral suppression during withdrawal from ethanol, nicotine, and cocaine. Prevented withdrawal‐induced anxiety‐like behavior. No rebound effects after ondansetron withdrawal.	Key Findings: Mecamylamine produced withdrawal‐associated place aversion. Ondansetron dose‐dependently attenuated this aversion; highest dose (0.1 mg/kg) nearly abolished effect. Ondansetron alone did not induce preference/aversion.
Methodological Flaws: Animal model; short duration; indirect behavioral outcomes	Methodological Flaws: Animal model; short nicotine exposure; focused on acute aversion, not craving/relapse
Zacny et al. [5]	West & Hajek [6]
Study Type: Human, inpatient, prospective, crossover, placebo‐controlled trial	Study Type: Human, randomized, double‐blind, placebo‐controlled trial
*N*: Exp 1: 7 smokers (4 M/3F, ~20 cig/day); Exp 2: 7 smokers (~27 cig/day)	*N*: 111 smokers who were enrolled in a smoking cessation program
Dose and Duration: Exp 1: Ondansetron 0.15, 0.3, 0.45 mg/kg IV (divided TID) over 24 h; Exp 2: Ondansetron 0.01, 0.02, 0.04 mg/kg IV (divided TID) over 24 h	Dose and Duration: Ondansetron 0.25 mg BID, started 2 weeks before the quit date and continued for a total of 4 weeks.
Key Findings: No effect of ondansetron on cigarettes smoked, expired CO, plasma nicotine/cotinine, or mood/craving. No dose–response trends.	Key Findings: Ondansetron did not reduce withdrawal symptoms or improve abstinence rates compared to placebo during the 4‐week treatment period
Methodological Flaws: Very small N; acute 24 h exposure only; inpatient non‐naturalistic setting; participants not motivated to quit; only acute dosing tested	Methodological Flaws: Relatively small sample size, short duration of follow‐up, dose or treatment period may have been insufficient to detect a clinically meaningful effect

More recently, studies have highlighted the insula, a cortical region integrating interoceptive, emotional, and cognitive functions, in nicotine craving, withdrawal, and smoking maintenance, making this region a promising target for cessation interventions [8]. A double‐blind, placebo‐controlled crossover study in 53 healthy subjects found that high doses of ondansetron (8, 16, and 24 mg) produced a dose‐dependent decrease in insula activation during a sensorimotor task, with the most pronounced reduction at 24 mg and milder effects at 16 and 8 mg [9]. Building on this, another study reported that high‐dose ondansetron (24 mg/day) significantly reduced compulsive behaviors in patients with obsessive‐compulsive disorder (OCD) who were concurrently taking serotonin reuptake inhibitors (SRIs); in contrast, no effect was observed in medication‐free participants [10]. Because OCD symptoms have been linked to increased insula activity, these findings suggest that ondansetron, particularly when combined with an SRI, may act synergistically to reduce insula activation [11].

Supporting the relevance of insula modulation to smoking cessation, multiple studies have shown that patients with lacunar infarcts involving the insula exhibit significantly higher rates of smoking cessation [12, 13]. Individuals with insula vascular lesions experienced reductions in cigarette cravings and withdrawal symptoms and were less likely to require nicotine replacement therapy compared to those with non‐insula cerebral vascular damage. Therefore, it is reasonable to suggest that the insula may be an important target for long‐term smoking cessation therapies.

Given the emerging evidence, we believe that a renewed investigation of ondansetron for smoking cessation is warranted. Prior studies were constrained by small sample sizes, subtherapeutic dosing, and short follow‐up. Recent findings suggest that high‐dose ondansetron reduces insula activity, a brain region linked to cigarette craving, and that its combination with SRIs reduces behaviors associated with insula activity. Beyond these neurophysiological observations, ondansetron's action as a selective 5‐HT3 receptor antagonist is particularly relevant to the neurobiology of addiction. The 5‐HT3 receptor is a ligand‐gated ion channel expressed in brain regions integral to reinforcement and craving, including the insula, prefrontal cortex, amygdala, and the nucleus accumbens within the mesolimbic dopamine system [14]. Because nicotine excites serotonergic projections from the dorsal raphe to the nucleus accumbens, antagonism at the 5‐HT3 receptor may reduce this serotonergic drive and thereby attenuate nicotine‐induced dopamine release, blunting the phasic dopamine response linked to reinforcement and craving [15].

Current clinical guidelines recommend varenicline, bupropion, and nicotine replacement therapy as first‐line treatments, often in combination with behavioral support, yet many patients relapse despite these options, and some are unable to tolerate them due to adverse effects [1, 2, 16]. High‐dose ondansetron, particularly in combination with an SRI, may therefore represent a promising adjunctive strategy for individuals who do not respond adequately to or cannot tolerate existing therapies. Prior research has shown that high‐dose ondansetron is generally safe and well tolerated, though constipation was a notable side effect in 52% of participants, warranting monitoring in future trials [10]. Ultimately, large‐scale, placebo‐controlled trials are needed to evaluate whether high‐dose ondansetron in combination with an SRI offers a more effective approach to improving smoking cessation outcomes.

## Author Contributions

Amanda Tam drafted the original manuscript and contributed to review and editing. Zoya Munsar drafted the original manuscript and contributed to review and editing. Douglas J. Opler provided conceptualization, supervision, and contributed to review and editing. All authors have read and approved the final version of the manuscript.

## Ethics Statement

The authors have nothing to report.

## Consent

The authors have nothing to report.

## Conflicts of Interest

The authors declare no conflicts of interest.

## Data Availability

The authors have nothing to report.

## References

[1] L. García‐Gómez , A. Hernández‐Pérez , V. Noé‐Díaz , J. A. Riesco‐Miranda , and C. Jiménez‐Ruiz , “Smoking Cessation Treatments: Current Psychological and Pharmacological Options,” Revista de Investigación Clínica 71, no. 1 (2019): 7–16, 10.24875/RIC.18002629. 30810545

[2] S. E. Jackson , J. A. McGowan , H. K. Ubhi , et al., “Modelling Continuous Abstinence Rates Over Time From Clinical Trials of Pharmacological Interventions for Smoking Cessation,” Addiction 114, no. 5 (2019): 787–797, 10.1111/add.14549. 30614586 PMC6492005

[3] B. Costall , B. J. Jones , M. E. Kelly , R. J. Naylor , E. S. Onaivi , and M. B. Tyers , “Ondansetron Inhibits a Behavioural Consequence of Withdrawing From Drugs of Abuse,” Pharmacology, Biochemistry, and Behavior 36, no. 2 (1990): 339–344, 10.1016/0091-3057(90)90414-d. 2141423

[4] T. Suzuki , Y. Ise , T. Mori , and M. Misawa , “Attenuation of Mecamylamine‐Precipitated Nicotine‐Withdrawal Aversion by the 5‐HT3 Receptor Antagonist Ondansetron,” Life Sciences 61, no. 16 (1997): PL249–PL254, 10.1016/s0024-3205(97)00745-5. 9353175

[5] J. P. Zacny , J. L. Apfelbaum , J. L. Lichtor , and J. G. Zaragoza , “Effects of 5‐hydroxytryptamine3 Antagonist, Ondansetron, on Cigarette Smoking, Smoke Exposure, and Mood in Humans,” Pharmacology, Biochemistry, and Behavior 44, no. 2 (1993): 387–391, 10.1016/0091-3057(93)90479-d. 8446670

[6] R. West and P. Hajek , “Randomised Controlled Trial of Ondansetron in Smoking Cessation,” Psychopharmacology 126, no. 1 (1996): 95–96, 10.1007/BF02246417. 8853223

[7] C. D. Cropp and M. L. Gora‐Harper , “Ondansetron Use for Smoking Cessation,” Annals of Pharmacotherapy 29, no. 10 (1995): 1041–1042, 10.1177/106002809502901016. 8845543

[8] M. F. Regner , J. Tregellas , B. Kluger , K. Wylie , J. L. Gowin , and J. Tanabe , “The Insula in Nicotine Use Disorder: Functional Neuroimaging and Implications for Neuromodulation,” Neuroscience and Biobehavioral Reviews 103 (2019): 414–424, 10.1016/j.neubiorev.2019.06.002. 31207255 PMC12931444

[9] E. R. Stern , R. Shahab , S. J. Grimaldi , et al., “High‐Dose Ondansetron Reduces Activation of Interoceptive and Sensorimotor Brain Regions,” Neuropsychopharmacology 44, no. 2 (2019): 390–398, 10.1038/s41386-018-0174-x. 30116006 PMC6300545

[10] E. R. Stern , K. A. Collins , L. B. Bragdon , et al., “Randomized Controlled Trial of the Effects of High‐Dose Ondansetron on Clinical Symptoms and Brain Connectivity in Obsessive‐Compulsive and Tic Disorders,” American Journal of Psychiatry 182, no. 3 (2025): 285–296, 10.1176/appi.ajp.20240294. 39876680 PMC12622368

[11] H. A. Berlin , E. R. Stern , J. Ng , et al., “Altered Olfactory Processing and Increased Insula Activity in Patients With Obsessive‐Compulsive Disorder: An fMRI Study,” Psychiatry Research: Neuroimaging 262 (2017): 15–24, 10.1016/j.pscychresns.2017.01.012. 28208068 PMC5373557

[12] A. Abdolahi , G. C. Williams , C. G. Benesch , et al., “Smoking Cessation Behaviors Three Months Following Acute Insular Damage From Stroke,” Addictive Behaviors 51 (2015): 24–30, 10.1016/j.addbeh.2015.07.001. 26188468 PMC4558299

[13] R. Suñer‐Soler , A. Grau , M. E. Gras , et al., “Smoking Cessation 1 Year Poststroke and Damage to the Insular Cortex,” Stroke 43, no. 1 (2012): 131–136, 10.1161/STROKEAHA.111.630004. 22052507

[14] J. Walstab , G. Rappold , and B. Niesler , “5‐HT(3) Receptors: Role in Disease and Target of Drugs,” Pharmacology & Therapeutics 128, no. 1 (2010): 146–169, 10.1016/j.pharmthera.2010.07.001. 20621123

[15] B. Chang , C. A. Daniele , K. Gallagher , et al., “Nicotinic Excitation of Serotonergic Projections From Dorsal Raphe to the Nucleus Accumbens,” Journal of Neurophysiology 106, no. 2 (2011): 801–808, 10.1152/jn.00575.2010. 21593391 PMC3154831

[16] US Preventive Services Task Force , “Interventions for Tobacco Smoking Cessation in Adults, Including Pregnant Persons: US Preventive Services Task Force Recommendation Statement,” JAMA 325, no. 3 (2021): 265–279, 10.1001/jama.2020.25019. 33464343

